# De Novo Kaposi Sarcoma in an HIV-Negative Liver Transplant Recipient With Ulcerative Colitis and Primary Sclerosing Cholangitis

**DOI:** 10.1155/crit/4699128

**Published:** 2024-11-12

**Authors:** Pavithra Ramakrishnan, Khalid Amin, Wolfgang Gaertner, Elizabeth S. Aby

**Affiliations:** ^1^Department of Medicine, University of Minnesota, Minneapolis, Minnesota, USA; ^2^Department of Pathology, University of Minnesota, Minneapolis, Minnesota, USA; ^3^Division of Colon and Rectal Surgery, University of Minnesota, Minneapolis, Minnesota, USA; ^4^Division of Gastroenterology, Hepatology and Nutrition, University of Minnesota, Minneapolis, Minnesota, USA

**Keywords:** IBD, Kaposi sarcoma, liver transplant, ulcerative colitis, vedolizumab

## Abstract

De novo or viral reactivation cancers are a major cause of morbidity and mortality in the solid organ transplant (SOT) population. Primary sclerosing cholangitis (PSC) is an aggressive disease which can lead to cholestatic liver damage and cirrhosis. PSC often cooccurs with inflammatory bowel disease (IBD). Here, we describe the case of a 28-year-old male with PSC along with poorly controlled IBD who underwent a liver transplant and developed colonic Kaposi sarcoma (KS). Our case highlights the importance of adequate pretransplant screening for endemic viruses, high clinical suspicion for KS in the setting of difficult-to-control colitis, and early multidisciplinary involvement.

## 1. Introduction

Primary sclerosing cholangitis (PSC) is a progressive cholestatic liver disease that leads to biliary stasis because of inflammation and fibrosis of intra- and extrahepatic biliary ducts. PSC is commonly associated with inflammatory bowel disease (IBD) [1]. The disease course of PSC can be heterogeneous but is often progressive and can lead to cirrhosis. PSC is also associated with bacterial cholangitis, cholangiocarcinoma, and colorectal cancer (CRC). It is estimated that 46% of patients presenting with PSC will require a liver transplant (LT) during their lifetime. Recurrent PSC is noted in up to 25% of patients posttransplant, most of which are diagnosed within 5 years [2]. Compared to age-matched controls, LT patients have a 2–3-fold higher overall cancer incidence [3]. A meta-analysis found that the incidence rate of CRC after LT for patients with PSC is 5.8 per 1000 person-years, which increases to 13.5 per 1000 person-years in the PSC-IBD subgroup who have an intact colon at the time of LT [4]. Oncogenic virus–related cancers caused by de novo exposure or reactivation of oncogenic viruses like human herpesvirus-8 (HHV-8), Epstein–Barr virus (EBV), and human papillomavirus (HPV) are seen in higher proportion in the immune-suppressed post-LT population. Surveillance, early detection, and recognition of atypical presentations of cancer are crucial to increase cancer survival. Herein, we present a case of isolated colonic Kaposi sarcoma (KS) in a post-LT patient with PSC-IBD and discuss current literature on this topic.

## 2. Case Presentation

**Figure 1:**
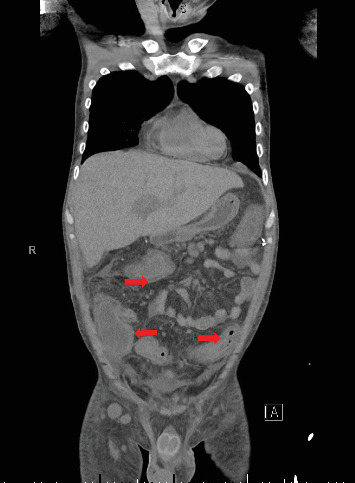
Computer tomography of chest abdomen pelvis coronal section showing severe pancolitis with loss of normal haustral colonic marking (indicated by red arrow).

**Figure 2:**
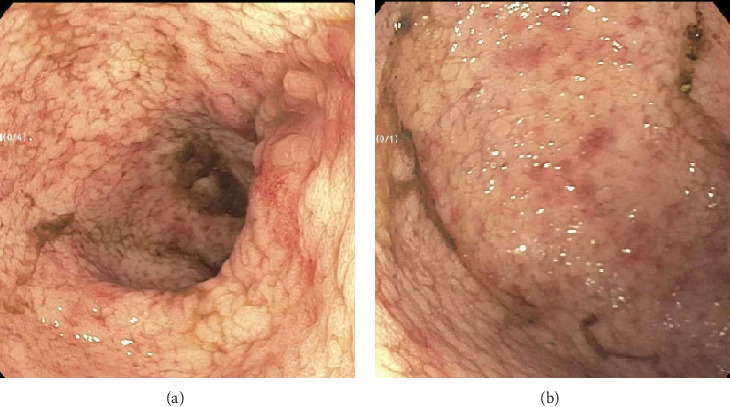
(a, b) Images taken during flexible sigmoidoscopy of the sigmoid colon showing altered vascularity.

**Figure 3:**
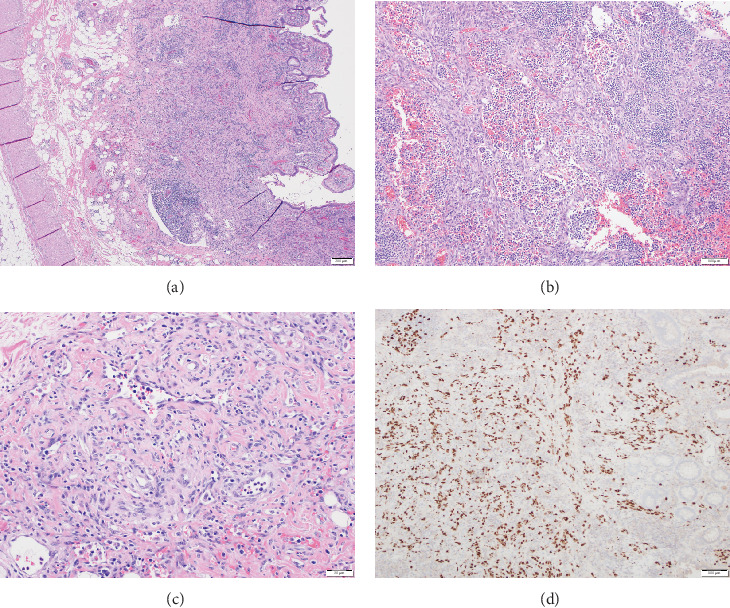
(a) 10X magnification hematoxylin and eosin stain (H&E) of colonic wall showing spindle cell tumor occupying the majority of mucosa and submucosal space. (b) 20X magnification H&E stain KS involving lymph node with effacement of normal nodal architecture (all submitted nodes were involved by the tumor). (c) 4X magnification H&E stain high power view of tumor composed of dilated irregular vascular channels surrounded by smaller vessels of varying caliber. (d) 10X magnification HHV-8 immune peroxidase stain depicting strong nuclear staining of tumor cells, supporting the diagnosis of KS.

**Figure 4:**
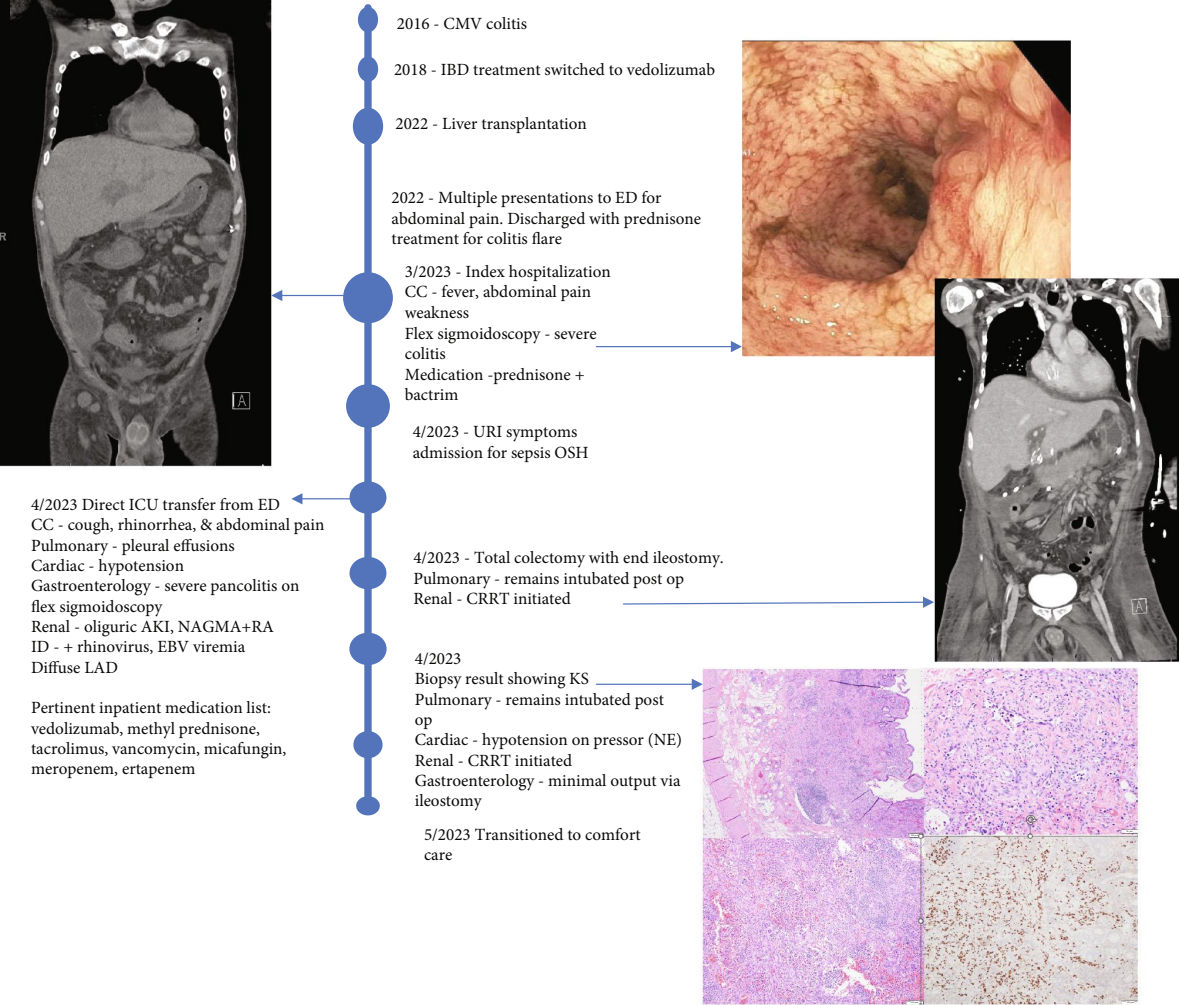
Timeline schema of the case outlining key events.

A 28-year-old male originally from Guatemala with a past medical history of congenital asplenia and a 19-year history of moderate to severe ulcerative pancolitis, cytomegalovirus (CMV) colitis, and PSC underwent LT with a seroconcordant CMV and EBV-matched positive donor. The posttransplant immunosuppression regimen was steroid induction, followed by tacrolimus and mycophenolate as maintenance. The patient was on infliximab 7 years pre-LT but transitioned to vedolizumab 4 years pre-LT given increasingly refractory colitis. Vedolizumab was initially held pre-LT but was resumed 4 months post-LT. Despite vedolizumab resumption, his colitis became increasingly refractory, requiring multiple prednisone courses. He suffered a second UC flare 6 months post-LT, requiring hospitalization. Infectious workup during his stay was negative for *Clostridioides difficile* and CMV. Colonoscopy assessment showed worsening active pancolitis with severe ulcerations in the rectum and sigmoid colon. Histologic examination at that time showed chronic colitis with moderate activity without dysplasia. HSV and CMV stains were negative. Notably, prior to the index hospitalization at our center, the patient had been on high-dose steroids consistently for the period of 1 month. Shortly after these episodes of worsening colitis, he was hospitalized for rhinovirus-associated severe septic shock. Computed tomography showed significant pancolitis and diffuse lymphadenopathy concerning posttransplant lymphoproliferative disorder (Figure 1). Flexible sigmoidoscopy upon hospital admission showed altered vascularity and friability throughout the rectosigmoid colon (Figure 2). Histologic examination demonstrated ulcerated mucosa with mixed inflammation and granulation tissue. HSV, CMV, and EBV stains were negative. PAS fungal stain was negative for organisms. There was no evidence of granuloma formation or dysplasia. High-dose parenteral steroid therapy was initiated in addition to his tacrolimus and vedolizumab. He developed an acute abdomen after 72 h and subsequently underwent emergent total abdominal colectomy with end ileostomy. The postoperative course was complicated by severe shock, worsening kidney injury, and encephalopathy. Colon histopathology demonstrated HHV-8-associated colonic KS with diffuse involvement of the submucosa, focal involvement of pericolic soft tissue, and lymph node involvement (Figure 3). There was evidence of background crypt glandular distortion and paneth cell metaplasia, compatible with a history of ulcerative colitis (UC). No dysplasia was noted. Serum HHV-8 PCR was 3,700,000 copies/mL. HIV was undetectable. No skin lesions were noted. In the setting of critical illness, KS-specific therapies were deferred. Given his metastatic KS and limited prognosis, the patient and family elected to pursue comfort care, and he passed away. The scheme of the patient's case is represented in Figure 4.

## 3. Discussion

KS was first described in 1872 by Moritz Kaposi as “idiopathic multiple skin pigmentation.” The causative agent was first discovered in 1994 as HHV-8 [5]. In addition to KS, HHV-8 also causes primary effusion lymphoma, multicentric Castleman disease, diffuse large B cell lymphoma, and inflammatory cytokine syndrome. The clinical presentation, diagnosis, and management of the non-KS entities are beyond the scope of this review but are well described elsewhere [5]. The four commonly described epidemiological forms of KS include AIDS-related, endemic, classic, or sporadic and iatrogenic (transplant or immune suppression–related). Solid organ transplant (SOT) patients are at a 60–200-fold higher risk of KS compared to the general population, with an incidence rate of 12.7 per 100,000 person-years in the LT population [6].

KS cases occur more commonly in HHV-8-endemic areas. Seroprevalence of HHV-8 has been found in up to 50% of the population in sub-Saharan Africa, 10%–40% in the Mediterranean, up to 20% in Asia, and about 30%–40% among the high-risk patients in Europe and the United States, which includes people living with HIV and men who have sex with men (MSM) [7]. When treating a diverse SOT patient population, it is important to consider the KS seroprevalence in their country of origin to inform pretransplant screening and surveillance testing.

In the HIV-negative population with IBD, the majority of KS has been reported in males, with poorly controlled UC and exposure to corticosteroids [8]. Common presentations include visceral KS without cutaneous lesions, which is often mistaken for IBD. Additionally, colonic KS changes occur at the submucosal level, followed by mucosal lesion development, which can often be mistaken for pseudopolyps [9]. High geographic seroprevalence of HHV-8 and the first year in the posttransplant period are reported to be risk factors for KS development within the SOT population [10]. Our patient developed KS within the first year post-LT. Posttransplant, the patient received the standard immunosuppression regimen at our center, which included steroid induction followed by taper, tacrolimus, and mycophenolate. Per protocol, his mycophenolate was weaned starting at 3 months, given no issues with rejection post-LT. In addition to the standard immunosuppression regimen post-LT, he was on vedolizumab, which causes further immunosuppression. No association between specific categories of immunosuppressive medications and the development of KS has been reported. Thus far, only one report of KS has been reported in an HIV-negative patient postkidney transplant [11]. Our case represents the first reported case of KS post-LT in a patient with IBD and PSC.

The 2019 American Society of Transplantation guidelines recommend considering pre-SOT HHV-8 screening for at-risk populations, which includes people with HIV, MSM, and donors and recipients from high seroprevalence areas [12]. HHV-8 serologic testing can be considered for screening in high-risk donors and recipients but must be interpreted with caution as there is variability based on assay characteristics and antigen preparations [12]. If an HHV-8 syndrome is suspected, the diagnosis of KS is made through a biopsy of the suspected site, followed by immunohistochemical statin and either in situ hybridization or viral PCR testing. Serological testing, with immunofluorescence assays, enzyme-linked immunosorbent assays, and western blot to detect latent and lytic phase antigens, has been shown to have poor performance in low seroprevalence populations [13]. Histological presentation of the disease can vary, but hallmark findings include the proliferation of small and irregular endothelium-lined spaces surrounding normal vessels and adnexal structures. Additionally, the presence of spindle cells with immunohistochemical staining for markers like CD 31, CD 34, ERG, and D2-40 is characteristic of KS [14]. KS management is mainly through complete or partial immune reconstitution. Oftentimes, immune suppression is adjusted by either reducing or eliminating calcineurin inhibitor use and adding a mammalian target of rapamycin (mTOR) inhibitor [12, 15]. mTOR is a well-established growth regulator important for cell proliferation and survival. Within the renal transplant population, the use of mTOR inhibitors has been thought to have direct antioncogenic effects and has been shown to be associated with a lower incidence of malignancy when used as a single therapy or in combination with calcineurin inhibitors [16]. The use of mTOR inhibitors in renal transplant recipients has been shown to cause regression of KS [17]. The mechanism behind this effect has not been fully elucidated but is suspected to be related to the reduction in vascular endothelial growth factor on endothelial cells [18]. Surgical resection, radiation therapy, and chemotherapy have been described as potential treatments [7].

The natural history of IBD post-SOT is poorly understood. A hypothesis for new IBD or worsening of preexisting disease is that most medications used for immune suppression in SOT such as corticosteroids, cyclosporine, and tacrolimus do not induce T lymphocyte apoptosis which is key to control IBD-related inflammation [19]. Loftus et al. observed that PSC-IBD may represent a unique form of IBD characterized by extensive colitis with rectal sparing and backwash ileitis [20]. PSC-IBD post-LT is a particularly vulnerable population with a 10-fold higher risk of CRC incidence than other LT recipients and a 20-fold higher risk compared to the general population [21]. Risk factors for CRC in this unique population include an over 10-year history of UC and extensive colitis, with the highest risk period being the first 2 years posttransplant, as seen in our case [4]. Consensus guidelines regarding the management of pretransplant IBD are lacking. According to the European Society of Organ Transplantation consensus statement published in 2023, pretransplant subtotal or total colectomy is strongly recommended for patients with fulminant colitis, active medication refractory colitis, and progressive colitis despite biologic therapy [22]. There is contradictory evidence regarding the role of pretransplant prophylactic colectomy and reduction of PSC recurrence risk, with the most recent evidence suggesting no benefit [23].

The management of IBD posttransplant can be challenging. When complications arise posttransplant, it is critical to involve a multidisciplinary team. In this case of colonic KS post-LT, the inclusion of colorectal surgeons, gastroenterologists, transplant hepatologists, and infectious disease specialists in the multidisciplinary discussions allowed for a comprehensive discussion of available treatment options to allow the patient and his family to choose what was in line with his goals.

## 4. Conclusion

In this case report, we present a case of colonic KS in a LT recipient with poorly controlled IBD. Our case highlights the consideration of pre-SOT screening for HHV-8 in donors and recipients from endemic regions to help stratify disease risk post-SOT. Additionally, in the subpopulation of patients with PSC and severe and/or poorly controlled IBD, pretransplant colectomy should be selectively considered after multidisciplinary discussion. The association between the duration or class of immunosuppressive medications and risk for KS in post-SOT patients is largely unknown. Three case reports have described colonic KS in patients with UC treated with vedolizumab [8, 24, 25]. A high index of suspicion is needed to identify colonic KS, as symptoms may mimic a severe UC flare. We believe this is the first reported case of colonic KS following LT in a patient with UC treated with vedolizumab. Our case raises important considerations about screening, consideration for pretransplant colectomy, choice of immune suppression, and early involvement of a multidisciplinary team.

## Data Availability

The authors have nothing to report.

## Nomenclature

CMV: cytomegalovirus
EBV: Epstein–Barr virus
HHV-8: human herpes virus 8
IBD: inflammatory bowel diseases
KS: Kaposi sarcoma
LT: liver transplant
PSC: primary sclerosing cholangitis
UC: ulcerative colitis
